# Timing of diuretic administration effects on urine volume in hospitalized patients

**DOI:** 10.3389/fphys.2023.1208324

**Published:** 2024-01-23

**Authors:** Katie S. McCullar, Sara Abbaspour, Wei Wang, Aaron D. Aguirre, M. Brandon Westover, Elizabeth B. Klerman

**Affiliations:** ^1^ Department of Neurology, Massachusetts General Hospital, Boston, MA, United States; ^2^ Division of Sleep Medicine, Harvard Medical School, Boston, MA, United States; ^3^ Division of Sleep and Circadian Disorders, Brigham and Women’s Hospital, Boston, MA, United States; ^4^ Department of Medicine, Massachusetts General Hospital, Boston, MA, United States

**Keywords:** chronotherapeutic, circadian medicine, diuretics, renal outcomes, inpatients

## Abstract

**Importance:** Some medications have effects that depend on the time of day they are given. Current knowledge of the time-of-day effects of specific medications in hospitalized patients with cardiovascular disease is very limited. In hospitalized patients, increased medication efficiency might reduce dose (and associated side effects) and/or the length of time in the Intensive Care Unit (ICU) or hospital–potentially improving patient outcomes and patient and family quality of life and reducing financial costs. We studied whether the time of day or night patients in Cardiac or Intensive Care Units receive a diuretic affects urine volume.

**Methods:** In this observational study, data were collected from 7,685 patients (63% male, 18 to 98 years old) admitted to one hospital’s Acute Care Cardiac units, Cardiac ICUs, Cardiac Surgery ICUs, and/or Non-cardiac ICUs who received intravenous furosemide (a diuretic), had measurements of urine volume, were hospitalized for ≥3 days between January 2016 to July 2021 and were older than 18 years. The outcomes of interest were urine volume normalized by the most recent (not older than 24 h) weight or body mass index (BMI), (i) in the hour after the time of diuretic administration, and (ii) when no diuretics were administered for the previous 3 h.

**Results:** We identified diuretic medication administration time 23:00–04:59 as a predictor of higher urine volume response. For patients without recent diuretic medication, higher urine volume was predicted 11:00–16:59 and 17:00–22:59. Other factors that affected urine volume response to the diuretic were sex, age, medication dose, creatinine concentration, diagnoses, and hospital unit.

**Discussion:** Time-of-day of medication administration may be a factor associated with increased medication efficiency. Randomized controlled trials should be conducted to quantify the relative effect of modifiable factors, such as time of medication administration, that may affect short- and longer-term outcomes.

## 1 Introduction

Circadian rhythms are physical, mental, and behavioral changes that display a period of approximately 24 h. These rhythms influence almost all areas of physiology, including the sleep-wake cycle, body temperature, blood pressure, and heart rate (Cederroth et al., 2019; Allada et al., 2021). While these rhythms are endogenously generated, the observed rhythm of the sleep-wake cycle, body temperature, blood pressure, heart rate, and other metrics is frequently modulated by external stimuli (e.g., light-dark cycle, meals, activity levels) (Klerman et al., 2022).

The circadian regulation of renal function is essential for maintaining fluid and electrolyte balance and blood pressure (Voogel et al., 2001; Firsov and Bonny, 2018; Johnston and Pollock, 2018). Glomerular filtration rate (GFR) is a measure of kidney filtering efficiency and exhibits diurnal variation, with higher GFR during the day compared to night (Koopman et al., 1989). Healthy individuals produce more urine during the day than at night, and there are circadian (i.e., endogenous) and time-of-day [“diurnal,” a combination of circadian rhythms and external stimuli (e.g., eating, posture changes, sleep/wake state) influences] in urinary sodium, potassium, and chloride excretion (Johnston and Pollock, 2018; Voogel et al., 2001; Stow and Gumz, 2011; Vagnucci et al., 1969; el-Hajj Fuleihan et al., 1997; McMullan et al., 2022; Min et al., 1966). Disruption of these rhythms is associated with hypertension, cardiovascular disease, and other adverse health outcomes (Firsov and Bonny, 2018).

Renal function and urine production are sensitive to endocrinological changes, and much of the circadian regulation of the kidneys occurs at a hormonal level (Solocinski and Gumz, 2015; Firsov and Bonny, 2018). Hormones, including cortisol, aldosterone, and renin, are required for healthy kidney function and exhibit a 24-hour rhythm (Solocinski and Gumz, 2015). In addition, aldosterone and anti-diuretic hormone (ADH) directly affect urine production and the secretion of these hormones changes across the 24-hour day (Asplund and Aberg, 1991; Solocinski and Gumz, 2015). Pharmacokinetics and pharmacodynamics are also altered by hormone levels (Kashuba and Nafziger, 1998; Beierle et al., 1999). Therefore, the effectiveness (and side-effects) of medication targeting any of these regulators of kidney function may have time-of-day variation (Ruben et al., 2019a; Ruben et al., 2019b).

Chronomedicine is an emerging field that explores biological rhythms and their impact on health (Allada et al., 2021). Specifically, this field aims to incorporate knowledge of biological rhythms to increase treatment effectiveness and decrease side effects (Ohdo, 2010; Ruben et al., 2019a; Ruben et al., 2019b; Allada et al., 2021; Kenig et al., 2021; Kramer et al., 2022). Chronomedicine has demonstrated clinical benefits in hypertension, hypercholesterolemia, cardiac arrhythmias, ischemic heart disease, cancer, diabetes, and other areas (Portaluppi and Lemmer, 2007; Glass-Marmor et al., 2007; Smolensky et al., 2007; Hermida and Ayala, 2009; Buttgereit et al., 2010; Ruben et al., 2019a; Ruben et al., 2019c; Gumz et al., 2022). The knowledge of medication time-of-day effects in hospitalized patients with cardiovascular disease is very limited. For hospitalized patients, there is tremendous potential benefit from any increased efficiency of medications. Another advantage of studying hospitalized patients is that there is a great source of accurately recorded data (e.g., medication dose and time, diet, frequently measured health outcomes such as vital signs or urine volume) to use for quantifying the impact of the timing of medication administration on patients’ health outcomes and the interactions of these variables. Adding a recommended time of day is a relatively low-cost change (i.e., not requiring the development of a new medication or other intervention) that can be implemented almost immediately. Time-of-day differences in the efficacy of these interventions could be important for understanding the physiology underlying these daily rhythms and potentially reducing doses given while achieving similar results.

In this study, we quantified the impact of the timing of diuretic medication administration and other covariates (e.g., sex, age, weight, medication dose, creatinine concentration, fluid intake, diagnoses, and hospital unit) on urine volume of hospitalized patients in Acute Care Cardiac Units and Intensive Care Units (ICUs). The source of the need to increase urine volume in patients in these hospital units may be heart or renal failure (with fluid retention), fluids given during surgery, or other causes. Diuretics (e.g., furosemide) are given to increase urine flow rate; they have a rapid onset of response within the first few minutes after intravenous (IV) diuretic administration and a half-life of ∼1 h. Our hypotheses were that, in this patient population, (i) the urine volume in the hour after a diuretic dose would vary by time-of-day, (ii) the urine volume per hour when no diuretic was recently administered would vary by time-of-day, and (iii) the time-of-day of maximum urine volume per hour with and without recent diuretic administration would differ.

## 2 Materials and methods

### 2.1 Data

The dataset was created from MassGeneralBrigham (MGB, Boston, MA, USA) electronic health records (EHR). The inclusion criteria were patients (i) admitted to Massachusetts General Hospital Acute Care Cardiac Units, Cardiac ICUs, Cardiac Surgery ICUs, and Non-cardiac ICUs, (ii) who received a discrete administration (“dose”) of furosemide through their IV, (iii) who had measurements of urine volume, (iv) were hospitalized for ≥3 days between January 2016 and July 2021, and (v) were age ≥18 years. Patients differed in the reasons for furosemide administration (e.g., heart failure, post-surgery).

Variables considered for analysis were selected based on expert knowledge and availability in EHR. Variables used were age, sex (female and male), weight, medication dose and time, lab test results (e.g., creatinine), fluid intake, medical condition/diagnosis (i.e., heart failure, acute kidney disease, chronic kidney disease, and cardiomyopathy), hospital unit, and urine volume and time. 0.8% of data samples were removed because of missing values of creatinine (0.6%) and weight (0.2%). Weights >200 kg or <40 kg were not used (0.8% of the data points). B-Type Natriuretic Peptide Test was initially considered for analysis, but there were not enough EHR data for this variable (95% missing values); therefore, it was not included in the final analyses. The outcomes of interest were urine volume rates normalized by the most recent (not older than 24 h) weight or BMI (i) in the hour after the time of medication administration and (ii) when no diuretics were received for the previous 3 h.

Analyses of some of these data using machine learning methods are available on MedRxiv at (Abbaspour et al., 2022).

The study was approved by The MGB Institutional Review Board without the need to obtain informed consent: # 2013P001024.

### 2.2 Pre-processing

The dataset was prepared by calculating the fluid intake rates and urine volume rates relative to medication administration time in hourly bins. All urine volume rates were normalized by weight or BMI (=weight in kg/height in m^2^). Medication administrations were included in the analyses when there were no other diuretic medications during the period 3 h before through 1 hour after that dose, and when there was no more than a 4-hour gap between consecutive urine volume measurements or fluid intake measurements. To identify predictors of urine volume response to medication, for each administered medication, urine volume 1 hour before medication administration, fluid intake rate 1 hour before medication administration, and urine volume in the hour after the time of medication administration were used for analysis. To identify predictors of urine volume without medication in the same patients, urine volume when no diuretic medications during the past 3 h were used for analysis. Time of day groupings were 23:00–04:59, 05:00–10:59, 11:00–16:59 or 17:00–22:59 based on recommendations in (Saver et al., 2023).

### 2.3 Statistical methods

Linear mixed-effects models were applied to examine the relationship between urine volume rate and preselected variables including sex, age, creatinine, admit diagnoses, hospital unit, medication dose, time of day of urine volume measurement, urine volume, and fluid intake rates during the past 1 hour. In the models: (i) time 23–05 h is the reference value for time; male is the reference value for sex, and Non-cardiac ICU is the reference value for hospital unit. (ii) no cardiomyopathy, heart failure, acute kidney disease, or chronic kidney disease are each the reference values for diagnosis. (iii) urine output 1 h before medication, fluid intake 1 h before medication, medication dose, age, and creatinine are continuous variables. Standardized coefficients were generated to enable the interpretability of the regression models. Urine rates were log-transformed to help meet the model assumptions. Residual plots were used for model diagnostics; no significant model violations were found. Analyses were performed using SAS (Cary, NC).

## 3 Results

There were 11,635 patients in total: 7,685 are in the dataset with medication administration; 11,540 in the dataset with no diuretics administered for the previous 3 h; and 7590 in both datasets. The dataset for analyses of urine volume after medication administrations includes 38,280 medication administrations in 7,685 patients during 8,305 hospital admissions; the dataset for analyses of urine volume when no diuretics were administered for the previous 3 h includes 11,540 patients during 13,078 hospital admissions. (Table 1).

**TABLE 1:** Number of individuals and (%) percent of total patients; Number of medication administrations and (%) percent of total medication administrations. Note, due to rounding, not all percentages sum to 100%.

Variables	With medication		Without medication	
	Number of individuals N(%)	Number of medication administration N(%)	Number of individuals N(%)	Number of urine collection N(%)
Sex				
Female	2,870 (37)	14,843 (39)	4,157 (36)	619,618 (36)
Male	4,815 (63)	23,437 (61)	7,383 (64)	1,123,498 (64)
Total	7,685 (100)	38,280 (100)	11,540 (100)	1,743,116 (100)
Admit Diagnosis				
Heart Failure	2051 (25)	8,691 (23)	3,462 (26)	559,149 (32)
Acute Kidney Disease	490 (6)	2,527 (7)	662 (5)	206,232 (12)
Chronic Kidney Disease	347 (4)	1,751 (5)	604 (5)	108,861 (6)
Cardiomyopathy	1,983 (23)	8,516 (22)	3,309 (25)	541,752 (31)
Other reasons for visit	5,471 (66)	25,602 (67)	8,463 (65)	941,952 (54)
Total	8,305 (100)	38,280 (100)	13,078 (100)	1,743,116 (100)
Hospital Unit				
Acute Care Cardiac Unit		2,764 (7)		258,474 (15)
Cardiac ICU		5,640 (15)		320,708 (18)
Cardiac Surgery ICU		14,290 (37)		527,408 (30)
Non-cardiac ICU		15,586 (41)		636,526 (37)
Total		38,280 (100)		1,743,116 (100)
Medication Time				
23-05H		5,991 (16)		397,480 (23)
05-11H		10,975 (29)		434,257 (25)
11-17H		13,060 (34)		446,251 (25)
17-23H		8,254 (21)		465,128 (27)
Total		38,280 (100)		1,743,116 (100)

### 3.1 Urine volume after diuretic medication

**FIGURE 1:**
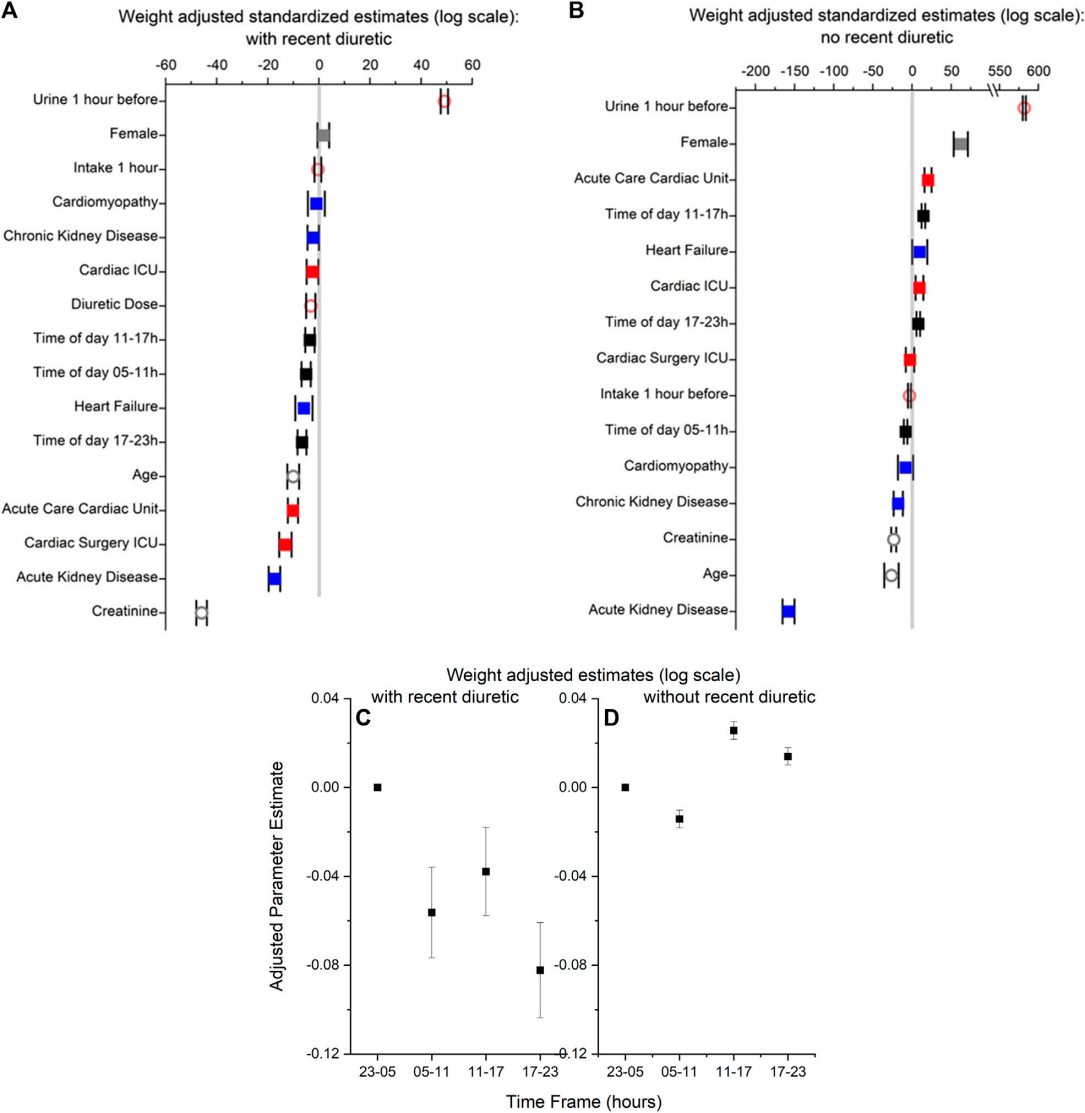
**(A,B)** Standardized parameter estimates with lower and upper bounds for features used to build regression models of weight-adjusted urine volume in 1 hour with (Panel **(A)**) and without recent diuretic medication (Panel **(B)**). In both panels, (i) time 23–05 h is the reference value for time of day (black symbols; male is the reference value for sex (grey symbols), and Non-cardiac ICU is the reference value for hospital unit (red symbols). (ii) no cardiomyopathy, heart failure, acute kidney disease or kidney disease are the reference values for diagnosis (blue symbols). (iii) urine output 1 h before medication (orange symbols), fluid intake 1 h before diuretic medication (orange symbols), diuretic medication dose (orange symbols), age (grey symbols), and creatinine (grey symbols) are continuous variables. Filled squares are for categorical variables; open circles are for continuous symbols. **(C,D)** Parameter estimates with lower and upper bounds from the regression models of weight-adjusted urine volume in 1 hour in panels A and B for the four time frames (i.e., 23–05 [reference time frame], 05–11, 11–17,17–23) for with (Panel **(C)**) and without recent diuretic medication (Panel **(D)**).

Nine features (sex, age, medication dose, creatinine, admission diagnoses, hospital unit, time of day of medication administration, urine volume, and fluid intake 1 hour before medication) were used to build a regression model for the estimation of weight-adjusted urine volume rate the hour after medication administration. The five most important predictors of urine volume the hour after medication administration were, in descending order: urine volume the hour before medication administration, creatinine, acute kidney disease, Cardiac Surgery ICU, and Acute Care Cardiac Unit (Figure 1A). The regression model with BMI-adjusted urine volumes is in Supplementary Figure S1A.

Diuretic medication administrations between 23:00 and 04:59 were associated with higher urine volumes than after diuretic medication administration 5:00–10:59, 11:00–16:59, or 17:00–22:59 (Figure 1C); higher urine volume 1 hour prior was also associated with higher urine volumes. Diagnoses of acute kidney disease or heart failure or admission to the Acute Care Cardiac Unit, Cardiac ICU, or Cardiac Surgery ICU were associated with a lower urine volume in response to diuretics. For the continuous variables, older age, higher creatinine concentrations, and higher medication doses were associated with lower urine volumes.

### 3.2 Urine volume without diuretic medication

Eight features (sex, age, creatinine, admit diagnoses, hospital unit, time of day of urine volume measurement, urine volume 1 hour prior, and fluid intake rates during the past hour) were used to build a regression model for the estimation of weight-adjusted urine volume rate without medication. The five most important factors to predict urine volume without medication were, in descending order: urine volume the hour before, acute kidney disease, age, creatinine, and chronic kidney disease (Figure 1B).

Time of day of urine collection between 11:00 and 16:59 or between 17:00 and 22:59 (compared to 23:00–04:59) (Figure 1D), female sex, urine volume 1 hour prior, and admission to the Acute Care Cardiac Unit or Cardiac Unit were associated with higher urine volume rates without medication. A diagnosis of acute kidney disease, chronic kidney disease, and/or cardiomyopathy and time of day of urine collection between 5:00 to 10:59 (compared to 23:00–04:59) were associated with a lower urine volume rate without medication. For the continuous variables, older age and higher creatinine concentrations were associated with lower urine volumes. The regression model with BMI-adjusted urine volumes is in Supplementary Figure S1B.

## 4 Discussion

In the present study, we quantified the effect of the time of day of a diuretic administration and other covariates on urine volume in hospitalized patients. We also quantified the effects of time of day and other covariates on urine volume without diuretic administration in the same population. Our results are consistent with prior publications that have described relationships between urine volume in response to a diuretic and age, sex, BMI, diagnosis of heart or kidney disease, and metrics of renal function (Aronson and Burger, 2016). The most novel finding is the time-of-day effects of IV furosemide on urine volume in hospitalized patients in ICUs, with a significantly larger effect of medication administration for doses between 11 pm and 5 am in a model that included fluid intake as a variable. Notably, this was a different time than the highest spontaneous (i.e., no recent diuretic medication) urine volume, which was during the day (11 am-5 pm and 5 pm- 11 pm). The differing effects of the specific hospital units on urine volume likely reflect the various comorbidities and reasons for admission in these populations. For example, patients in the Non-cardiac ICU may respond more vigorously to a given diuretic dose than those in the Cardiac ICU (Figure 1A), given the generally better cardiac or renal function in these patients. Our analysis of this data set using machine learning techniques and adjustment by BMI (not weight) (Abbaspour et al., 2022) also found greater response to diuretics between midnight and 6 am, with male sex, in some hospital units (e.g., Non-cardiac ICU), with younger age, and with lower creatinine levels; lower response to diuretics was associated with female sex, some diagnoses (heart failure, acute kidney disease, chronic kidney disease), and some hospital units. Further investigation of specific populations will be needed to elucidate these differences fully.

Potential mechanisms underlying these time-of-day effects include (i) time-of-day variation in kidney response to fluid volume and/or diuretics; (ii) time-of-day variation in metabolism of the diuretics; (iii) time-of-day variation in cardiac function and blood pressure impacting renal perfusion and filtration rates; (iv) time-of-day variation in hormone levels impacting renal function.

Chronomedicine aims to incorporate knowledge of biological rhythms in clinical practice to better inform care in both preventative and curative approaches (Roenneberg et al., 2022). Many healthcare professionals across specialties are beginning to consider chronobiology in their diagnosis and treatment administration. Expanding our understanding of time-of-day effects may be used to better define relevant physiology and improve clinical care in outpatient and inpatient populations. The availability of data from EHR (including telemetry) will enable additional studies of physical signs, symptoms, and responses to interventions in inpatient populations (e.g., (Ruben et al., 2019d; Mahdi et al., 2019; Beyer et al., 2021)). For example, altering the time of an intervention to increase efficacy would be a relatively low-cost and scalable change in practice: for this specific drug, administering furosemide between 11 pm and 5 am in individuals who can tolerate a urinary catheter would be an option. These findings of time-of-day effect of a diuretic in patients in ICUs are consistent with other work - mostly from outpatient studies - about time-of-day variation in response to medications (e.g., (Kashuba and Nafziger, 1998; Beierle et al., 1999; Ohdo, 2010; Ruben et al., 2019a; Ruben et al., 2019b; Ruben et al., 2019c; Kenig et al., 2021; Kramer et al., 2022)).

A limitation of this work is that the data are collected from a retrospective observational study, and studies of this nature may have biases. Since the data used were from hospital records, we did not have reliable data about physiological conditions that may have affected the body clock of the patients, including working night or rotating shifts, recent jet lag, sleeping problems, psychiatric illness, light exposure, genetics, or chronotype. In reference to the present study, potential biases may be related to interactions of diagnosis and timing or amount of medication administration and the impact of the timing and structure of the clinical staffing model and decision-making. For example, clinical rounding by medical staff multiple times a day compared to once per day may impact dosing adjustments across the day. Further, the environment of the hospital units (e.g., constant light conditions) and some patient procedures (e.g., sedation) in which these data were collected would be expected to affect underlying circadian rhythms. Interactions between diuretics and other drugs may influence the efficacy of diuretics; a limitation of the present study is that we did not adjust for this potential effect. These potential biases should be considered when designing a randomized controlled trial of the effects of the time of diuretics on different physiological outcomes. Randomized clinical trials, with collection of additional metrics (e.g., of cardiovascular and renal function and circadian rhythmicity) relevant to the underlying physiology, should be performed to further test our hypotheses of time-of-day influences on immediate drug effects, longer-term effects on multiple metrics of patient health and quality of life, length of stay in ICUs and in-hospital total, and financial costs. For hospitalized patients, administering medications at any time of day or night should be feasible and, if proven more effective, can be implemented almost immediately, making this an enticing and accessible avenue to optimize patient care. Basic science studies should also be done to better define physiology and develop new treatments. Education for healthcare providers (e.g., physicians, nurses, and pharmacists) about time-of-day effects and variation in clinical metrics should also be implemented.

In summary, our results demonstrate that younger age, lower creatinine concentration, and time of day of medication administration (11:00 pm–05:00 am) are associated with a significantly higher predicted urine volume response to a diuretic in these hospitalized patients. Adding a recommended time of day is a low-cost change (i.e., not requiring the development of a new medication or other intervention) that can be implemented almost immediately. Further, this study demonstrated that female sex, younger age, lower creatinine, and time of day (11:00–16:59 and 17:00–22:59) are associated with a significantly higher predicted urine volume without medication in these hospitalized patients. Time-of-day differences in the efficacy of interventions are important for understanding the underlying basic science and may lead to reduced doses of medication required to achieve similar results.

## Data Availability

The data analyzed in this study is subject to the following licenses/restrictions: Detailed data access will require institutional review board approval and a data use agreement with Mass General Brigham. Requests to access these datasets should be directed to EK, ebklerman@hms.harvard.edu.
