# Supplementary material for: Timing of diuretic administration effects on urine volume in hospitalized patients
Source: Front Physiol. 2024 Jan 23;14:1208324. doi: 10.3389/fphys.2023.1208324 (PMC10844419; doi:10.3389/fphys.2023.1208324)
Supplement: Supplementary file 1 [file DataSheet1.docx]

Timing of Diuretic Administration Effects on Urine Volume in Hospitalized Patients

Katie S McCullar^1,2**^, Sara Abbaspour PhD ^1,2**^, Wei Wang^2,3^, Aaron D. Aguirre MD PhD ^4^, M. Brandon Westover MD PhD ^1,5^, Elizabeth B. Klerman MD PhD ^1,2,3*^

^1^ Department of Neurology, Massachusetts General Hospital. Boston MA, USA

^2^ Division of Sleep Medicine, Harvard Medical School. Boston MA, USA

^3^ Division of Sleep and Circadian Disorders, Brigham and Women’s Hospital, Boston MA, USA

^4^ Department of Medicine, Massachusetts General Hospital. Boston MA, USA

^5^ Department of Neurology, Beth Israel Deaconess Hospital, Boston MA, USA

**** joint first authors**

*** Correspondence:**Elizabeth B Klerman MD PhD
ebklerman@hms.harvard.edu

Keywords: chronotherapeutic, circadian medicine, diuretics, renal outcomes, inpatients

# Supplementary Material

Data were also analyzed adjusted by BMI, instead of weight.

## Urine volumes after diuretic administration.

The same nine features were used to build a regression model for the estimation of BMI-adjusted urine volume rate the hour after medication administration as for the weight-adjusted volume rate. (Figure S1A).

Diagnoses of acute or chronic kidney disease or heart failure, or admission to the Acute Care Cardiac Unit, or Cardiac Surgery ICU, and female sex were associated with a lower urine volume in response to diuretics. Medication administrations between 23:00 and 04:59 and urine volumes one hour prior were associated with higher urine volumes. For the continuous variables, older age, higher creatinine concentrations, and higher medication doses were associated with lower urine volumes. These data are consistent with our analysis using machine learning techniques (1).

## Urine volume without diuretic medication

The same eight features (sex, age, creatinine, admit diagnoses, hospital unit, time of day of urine volume measurement, urine volume the hour prior, and fluid intake rates during the past hour) were used to build a regression model for the estimation of BMI-adjusted urine volume rate without medication as for the weight-adjusted volume rate. (Figure S1B).

Urine volume one hour prior, admission to the Acute Care Cardiac Unit or Cardiac Uni, and heart failure were associated with higher urine volume rates without medication. A diagnosis of acute kidney disease, chronic kidney disease, and/or cardiomyopathy and time of day of urine collection between 5:00 to 10:59 were associated with a lower urine volume rate without medication. For the continuous variables, female sex, older age and higher creatinine concentrations were associated with lower urine volumes.


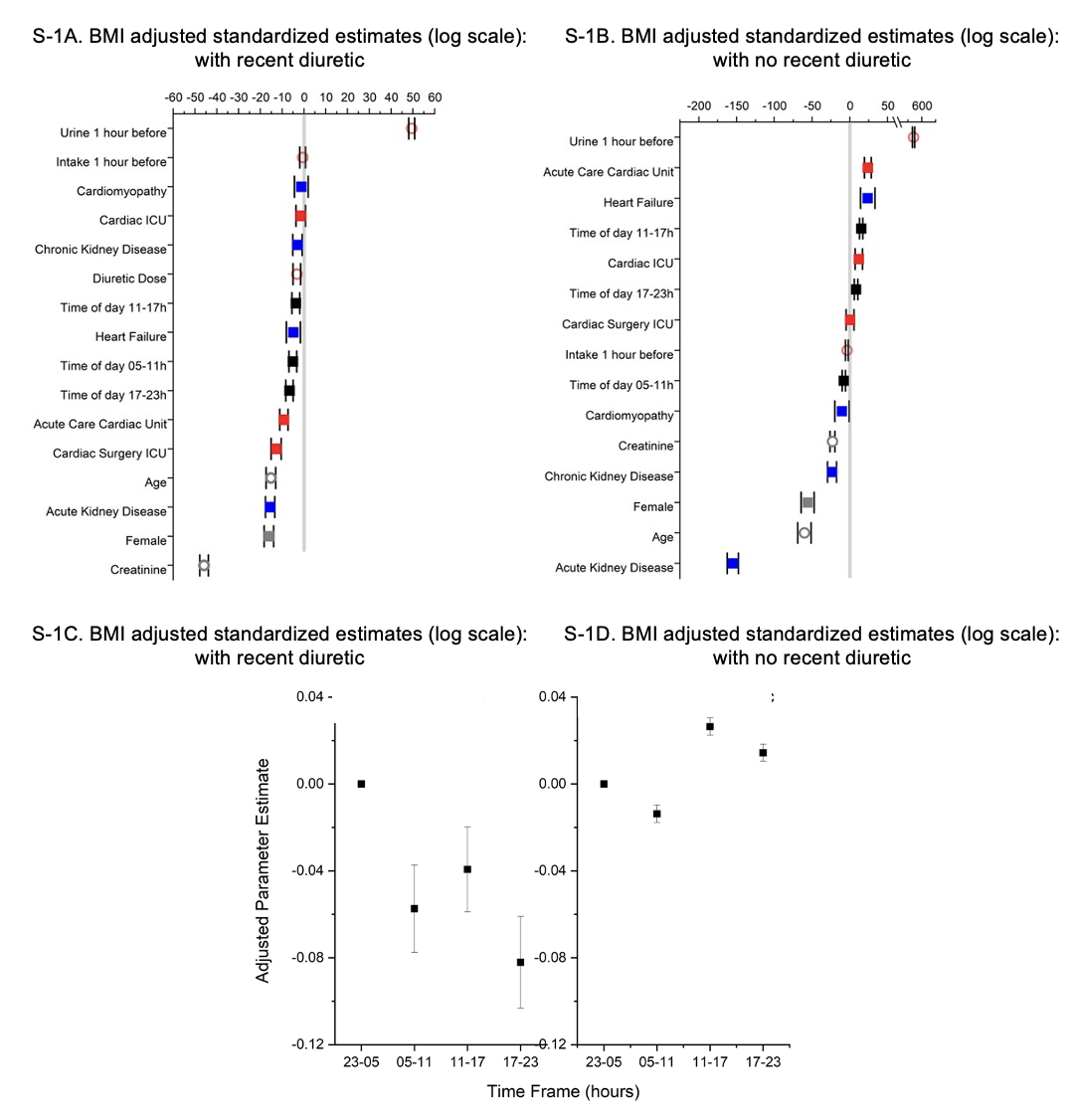


## Figure legends:

Figures S1A-S1D. As in Figures 1A-D except normalized by BMI instead of by weight.

## References:

Abbaspour S, Aguirre AD, Westover MB, Klerman EB. The Effects of the Timing of Diuretic Administration and Other Covariates on Urine Volume in Hospitalized Patients [Internet]. Cardiovascular Medicine; 2022 Dec [cited 2023 Oct 8]. Available from: http://medrxiv.org/lookup/doi/10.1101/2022.12.27.22283947
